# Immature platelet fraction and reticulated platelet parameters as potential indicators of bone-marrow-related platelet production in dogs and cats: A scoping review

**DOI:** 10.1371/journal.pone.0358333

**Published:** 2026-09-30

**Authors:** Mahmood Ahmadi-hamedani, Kimia Satari

**Affiliations:** 1 Department of Clinical Sciences, Faculty of Veterinary Medicine, Semnan University, Semnan, Iran; 2 Student Research Committee, Faculty of Veterinary Medicine, Semnan University, Semnan, Iran; Mayo Clinic Rochester: Mayo Clinic Minnesota, UNITED STATES OF AMERICA

## Abstract

**Background:**

Evaluating bone marrow is an invasive and expensive procedure that frequently necessitates sedation or anesthesia in dogs and cats. The immature platelet fraction (IPF(and related immature or reticulated platelet parameters, has been suggested as a non-invasive alternative for assessing megakaryocytic activity. However, a comprehensive assessment of the depth and balance of evidence across the two most prevalent companion animal species has not been conducted previously.

**Objective:**

Aim of this scoping review was: (1) to map and classify primary studies that report on IPF or reticulated platelets in the context of thrombopoietic activity or bone-marrow activity in dogs and/or cats, (2) to classify the subjects, assays, reference criteria, clinical information, findings etc; and (3) to highlight principal evidence gaps and identify if the evidence generated is adequate and comparable for the systematic review or for a future meta-analysis.

**Methods:**

Following the Population-Concept-Context (PCC) framework and the PRISMA Extension for Scoping Reviews (PRISMA-ScR guidelines, an exhaustive search was conducted in PubMed/MEDLINE, Web of Science, Scopus, and CAB Abstracts. The parameters of immature and reticulated platelets have been examined in relation to bone marrow and platelet status. Records were reviewed to ensure eligibility, and a standard data sheet was used to extract data.

**Results:**

Of 35 records identified, 10 met the eligibility criteria. Nine of the ten included studies reported data only in dogs; a single retrospective study reported data in both dogs and cats, and no study addressed cats exclusively. Only one study used a direct bone-marrow reference standard (megakaryocyte cytology); the remainder relied on indirect or clinical classification.

**Conclusions:**

Evidence for IPF/reticulated platelets as a bone-marrow marker is considerably more developed in dogs than in cats. Feline data are essentially limited to a single retrospective analyzer study. Given the small number of studies and marked methodological heterogeneity, a full systematic review with meta-analysis is not currently feasible; a scoping review is the appropriate and informative synthesis at this stage.

## 1. Introduction

Thrombocytopenia is one of the most common hematologic abnormalities encountered in small-animal practice, and distinguishing central (bone marrow-derived) from peripheral causes has direct implications for diagnostic work-up and treatment [1]. Direct assessment of marrow megakaryocytic activity traditionally requires bone marrow aspiration or core biopsy. These invasive procedures require sedation or general anesthesia, and are not always readily accepted by owners or easily repeated for monitoring purposes. The immature platelet fraction (IPF) and the related concept of reticulated platelets represent young, RNA-containing platelets recently released from the bone marrow; their proportion in circulation is thought to reflect the rate of thrombopoiesis, analogous to the reticulocyte count for erythropoiesis [2]. In human medicine, IPF is a validated, automated parameter used to differentiate hypoproliferative from destructive or consumptive thrombocytopenia. Its extension to veterinary hematology is more recent. It has been implemented on a range of analyzer platforms, including the Sysmex XN-1000V, Sysmex XT-2000iV, and ADVIA 2120, as well as by manual flow cytometry [2–5]. Despite growing interest, it remains unclear whether this literature has developed evenly across the two most common companion-animal species. Because sample volume, platelet clumping behavior, and disease epidemiology differ substantially between dogs and cats, findings from one species cannot be assumed to generalize to the other. A priori, a scoping review design was chosen due to the desire to map the scope, features, analytical methods, reference standards, clinical scenarios, and evidence gaps in the existing literature on IPF and its associated platelet parameters in dogs and cats. Specifically, the goals were to identify and describe characteristics of how evidence has been generated across species, analytical platforms, patient populations, and reference standards rather than to quantify an overall diagnostic effect. As a secondary outcome, a possibility of a future systematic review and/or meta-analysis could be inferred from the resulting evidence map.

## 2. Methods

### 2.1. Protocol and framework

This review used the framework outlined by Arksey and O’Malley [6] with the PRISMA-ScR flow diagram illustrating the study selection process (Fig 1) [7]. To address the research question, we applied the Population-Concept-Context (PCC) method (Table 1), a systematic method favored for scoping reviews. A priori, our review method was fixed, with the aim of charting the range and nature of research undertaken, the analytical methods applied, the use of reference standards and identification of gaps in this body of research; estimation of a single diagnostic accuracy measure was not desired.

**Fig 1:**
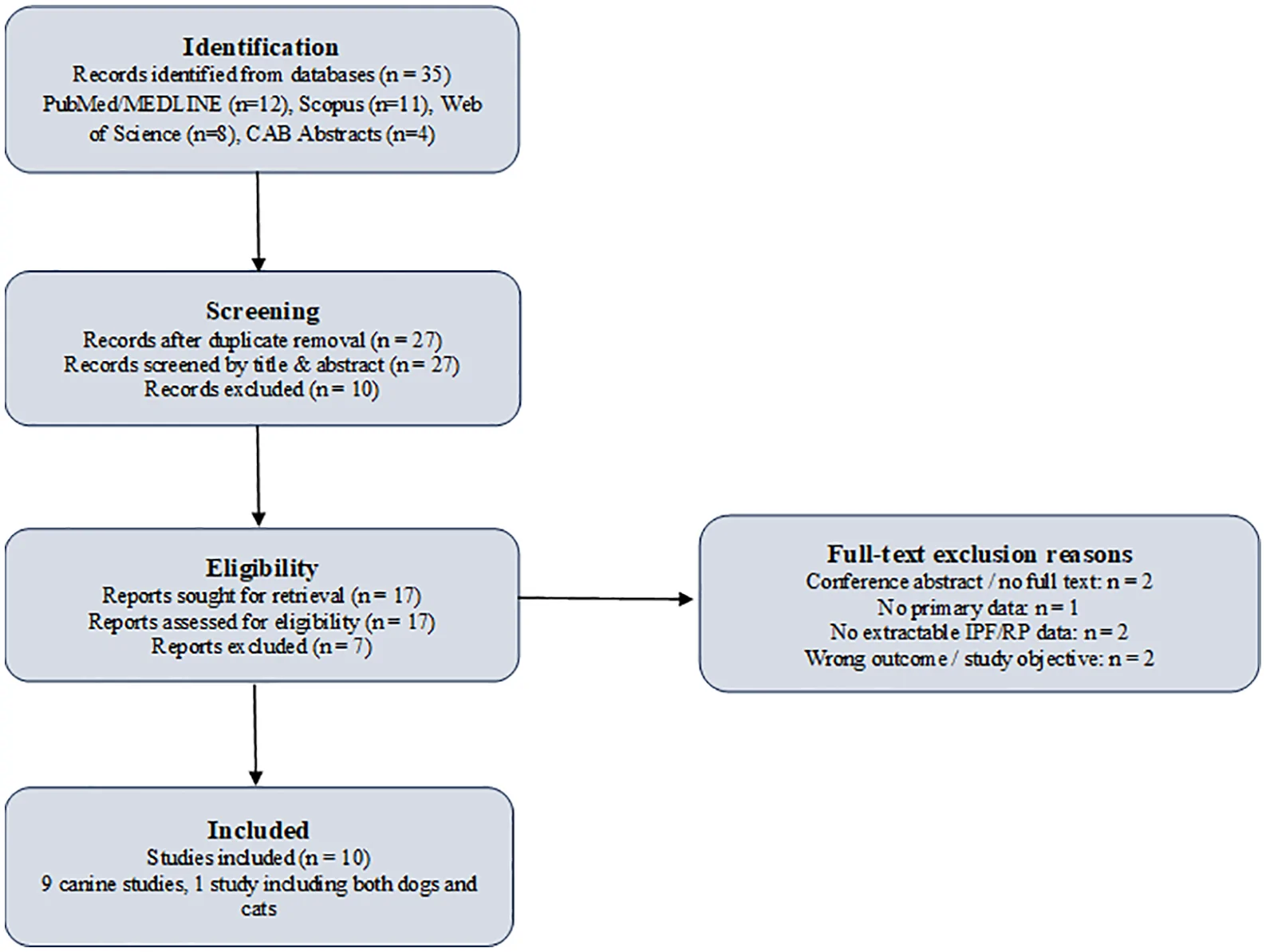
PRISMA-ScR flow diagram of study selection.

**Table 1:** Population-Concept-Context (PCC) framework used in this scoping review.

Component	Definition for this review
Population (P)	Dogs and cats – healthy animals and clinical patients (with thrombocytopenia, marrow disease, or other hematologic disorders)
Concept (C1)	Immature Platelet Fraction (IPF) and related parameters (Absolute Immature Platelet Fraction; A-IPF, Platelet-Fluorescence channel; PLT-F, reticulated platelets) as a laboratory marker
Context (C2)	Non-invasive assessment of megakaryocytic activity/ bone marrow function, including differentiation of central vs. peripheral thrombocytopenia

The protocol was later registered (retrospectively) via Zenodo (https://doi.org/10.5281/zenodo.21262147).

While this retro-registration is a limitation of this protocol, our eligibility criteria, search strategies and data extraction form were determined prior to any of the retrieved studies having been processed to their full text and were fixed thereafter.

### 2.2. Eligibility criteria

**Inclusion Criteria:** IPF refers to the percentage of immature platelets reported by automated hematology analyzers. Absolute immature platelet fraction (A-IPF or IPF#) refers to the absolute number or concentration of immature platelets. PLT-F refers to the platelet fluorescence channel or fluorescence-based platelet measurement used by specific analyzer platforms and should not be treated as synonymous with reticulated platelets. Reticulated platelets (RP or r-PLT) refer to RNA-containing immature platelets identified primarily by fluorescence-based methods such as thiazole-orange flow cytometry or analyzer-specific fluorescence methods. Because terminology and measurement procedures vary between analyzer platforms, these parameters were extracted and reported according to the terminology used in each original study. To address the review question, eligible studies were required to provide data relevant to thrombopoiesis, platelet production, thrombocytopenia classification, megakaryocytic activity, or the potential use or interpretation of immature or reticulated platelet parameters as indicators of bone-marrow-related platelet production. Studies did not need to include direct bone-marrow examination to be eligible, because an objective of this scoping review was to map the breadth of evidence supporting the biological and clinical interpretation of these platelet parameters, including studies providing indirect evidence such as reference intervals, analytical validation, method comparison, or association with thrombocytopenic states. Studies using a direct bone-marrow reference standard were considered to provide direct biological linkage to marrow activity and were identified separately during data charting. For final inclusion, the full-text report of an eligible primary study had to be available for assessment. Records available only as conference abstracts or without an accessible full-text primary research report were excluded at the full-text eligibility stage.

### 2.3. Information sources and search strategy

A comprehensive literature search was conducted using four electronic databases: PubMed/MEDLINE, Web of Science, Scopus, and CAB Abstracts. The literature search was conducted iteratively over approximately two months, with the final database searches completed on 5 July 2026. The search covered records from database inception to the date of the final search.

The search strategy was developed to identify studies investigating immature platelet fraction (IPF), reticulated or other immature platelet parameters in dogs and cats, particularly in relation to thrombopoiesis, thrombocytopenia, megakaryocytic activity, or bone marrow activity.

Search terms were developed based on terminology identified in the relevant veterinary and biomedical literature and included terms referring to immature platelet populations, analyzer-specific platelet parameters, and the target animal species. Database-specific search strategies were adapted according to the search syntax and indexing structure of each database S1 File.

PubMed/MEDLINE:

(“immature platelet fraction” OR “immature platelet” OR IPF OR “PLT-F” OR “A-IPF” OR “reticulated platelet”) AND (dog OR canine OR cat OR feline) AND (“bone marrow” OR megakaryocyte OR thrombopoiesis OR thrombocytopenia)

Web of Science:

TS=(“immature platelet fraction” OR “immature platelet” OR IPF OR “PLT-F” OR “A-IPF” OR “reticulated platelet”) AND TS=(dog OR canine OR cat OR feline) AND TS=(“bone marrow” OR megakaryocyte OR thrombopoiesis OR thrombocytopenia)

Scopus:

TITLE-ABS-KEY (“immature platelet fraction” OR “immature platelet*” OR IPF OR “PLT-F” OR “A-IPF” OR “reticulated platelet”) AND TITLE-ABS-KEY (dog OR canine OR cat OR feline) AND TITLE-ABS-KEY (“bone marrow” OR megakaryocyte OR thrombopoiesis OR thrombocytopenia)

CAB Abstracts:

(“immature platelet fraction” OR “immature platelet” OR IPF OR “PLT-F” OR “A-IPF” OR “reticulated platelet”) AND (dog OR canine OR cat OR feline) AND (“bone marrow” OR megakaryocyte OR thrombopoiesis OR thrombocytopenia)

The search conducted retrieved a total of 35 results: 12 from the PubMed/MEDLINE search, 11 from the Scopus search, eight from the Web of Science Core Collection and four from the CAB Abstracts search. No alternative hand-searching method, search of conference proceedings, or forward or backward citation tracking search was employed. Therefore, the entire 35 search results appearing in the PRISMA-ScR flow diagram arose from the four electronic searches included here.

### 2.4. Study selection

Based on the eligibility criteria outlined in Section 2.2, the 27 unique records were screened by two reviewers, who independently reviewed both the titles/abstracts and the full texts. There were no differences of opinion between the reviewers, nor any need for arbitration by any third party.

### 2.5. Data charting and synthesis

Data extraction and charting (Table 3) were performed by one reviewer and cross-checked by a second reviewer for accuracy S2 **and S3 files**. Data collected for all eligible studies (where applicable) included: study organism, study design, analytical or measurement techniques applied, parameters reported, major quantitative and/or qualitative findings, and application of a direct bone marrow reference standard. The findings are reported in a narrative synthesis, similar to that used in a scoping review. This approach accounts for potential variations across different platforms, analyzer standards, and results. For each included study, the relationship of the investigated platelet parameter to thrombopoiesis or bone-marrow activity was categorized as direct or indirect according to the presence or absence of a direct bone-marrow reference standard. Where a predefined data item was not explicitly reported in the full text, it was recorded as “not reported” and was not inferred.

## 3. Results

### 3.1. Study selection

The PRISMA-ScR flow diagram was used to summarize the study selection process (Fig 1). A total of 35 records were identified through comprehensive searches of PubMed/MEDLINE (n = 12), Scopus (n = 11), Web of Science (n = 8), and CAB Abstracts (n = 4). After removal of 8 duplicates, 27 unique records were available for title and abstract screening. At this point, 10 records were clearly irrelevant and excluded, leaving 17 full-text articles to be screened for eligibility. Seven of these full-text articles were excluded for the reasons presented in Table 2. Conference abstracts identified through the database searches were retained during screening and excluded at the full-text eligibility stage when they did not meet the predefined eligibility criteria. Thus, 10 original studies were included in the review, met all the eligibility criteria, and were charted in full (Section 3.3).

**Table 2:** Characteristics and reasons for exclusion of full-text articles assessed but not included (n = 7).

Author, Year [Ref]	Title (short)	Reason for exclusion
LeVine, 2025 [8]	Where did all the platelets go? Highlights of the Consensus statement on diagnosis of ITP in dogs and cats	Conference proceedings/abstract; no full-text primary data available
LeVine et al., 2024 (ACVIM) [9]	ACVIM consensus statement on treatment of ITP in dogs and cats	Review/consensus article — no primary data
Bachman et al., 2015 [10]	Modified PAIgG assay in thrombocytopenic dogs	No extractable IPF/reticulated platelet data
Franca et al., 2018 [11]	Surface immunoglobulins in dogs with Rangelia vitalii	No extractable IPF/reticulated platelet data
Martinez-Caro et al., 2025 [12]	Cryoglobulinemia + Leishmania interference with analyzers	Wrong outcome/study objective (case report; analyzer-interference focus)
Byun et al., 2026 [13]	Two companion canines with SFTS virus	Wrong outcome/study objective (case series; incidental IPF mention)
Jensen et al., 2021 [14]	Reticulated platelets in cats with cardiovascular disease	Conference abstract — no extractable full-text data

### 3.2. Excluded records

Of the 17 full-text articles assessed for eligibility, 7 were excluded S2 File. The most common reasons were review or consensus articles without primary data (n = 2) and a lack of extractable IPF/reticulated platelet data (n = 2), followed by an incorrect outcome or study objective (n = 2) and a conference abstract format without full text (n = 1). Table 2 lists each excluded record together with its specific reason for exclusion [8–14] (Table 2).

### 3.3. Characteristics and findings of included studies

The 10 included studies are summarized in Table 3 and were published between 2001 and 2026 [1–5, 15–19]. Feline evidence was limited to the retrospective study by Perez-Ecija et al. [15], which included 726 cats and 3,281 dogs. No feline-only study met the eligibility criteria. The included studies evaluated immature or reticulated platelet parameters across different analytical platforms and clinical contexts, including reference-interval establishment, thrombocytopenia classification, analytical validation, and method comparison. The parameters assessed were not identical across studies. Depending on the study and analytical platform, investigators evaluated reticulated platelets (RP or r-PLT), immature platelet fraction (IPF), absolute immature platelet fraction (IPF#), or platelet fluorescence (PLT-F)-related parameters. These measures were therefore considered related but not interchangeable platelet parameters and were not treated as equivalent analytical measures in the present review.

**Table 3:** Summary of study characteristics, methods, and findings for included studies (n = 10).

Author, Year [Ref]	Species; sample size	Study design	Analyzer/ Method	^a^Parameter	Reference interval/ Threshold/ Diagnostic statistics	Key findings relevant to thrombopoiesis or marrow activity	^b^BM relationship	Main study limitation
**Perez-Ecija et al., 2024 [15]**	Dogs (n = 3,281); Cats (n = 726)	Retrospective diagnostic study	Sysmex XN-1000V; PLT-F channel	IPF (%)	Dogs RI: 0.5–8%; cats RI: 1–40.3%. CEN cutoff: < 6.9% in dogs and <13.6% in cats after exclusion of pseudo-thrombocytopenia. Sensitivity/specificity: 95.1%/94.6% in dogs and 94.0%/85.3% in cats.	IPF differed significantly among thrombocytopenia subtypes in both species and was proposed to distinguish central from peripheral thrombocytopenia	Indirect	Retrospective design; very wide feline RI; direct marrow examination was not used
**Jornet-Rius et al., 2023 [3]**	Dogs; healthy RI cohort **n = 69**	Analytical validation/ reference-interval study	Sysmex XN-1000V; PLT-F channel	IPF (%), IPF# (absolute IPF), PLT-F	IPF RI: **1.24–10.42%** in all healthy dogs; **1.04–6.85%** without platelet aggregates. IPF#: **1.68–25.56 × 10⁹/L** overall; **1.31–20.59 × 10⁹/L** without aggregates	Established analytical performance and preliminary canine RIs; platelet aggregation affected IPF and IPF# values	Indirect	Preliminary RI study; platelet aggregation substantially influenced measurements
**Zmigrodzka et al., 2014 [16]**	Dogs; **n = 72** (30 controls, 42 thrombocytopenic)	Cross-sectional clinical study	Flow cytometry; thiazole orange staining	Reticulated platelets (%)	No diagnostic cut-off reported	RP% was significantly higher in thrombocytopenic dogs; findings supported preserved/competent thrombopoiesis in breed-related thrombocytopenia	Indirect	Breed-related thrombocytopenia population; no direct marrow quantification
**Wilkerson et al., 2001 [17]**	Dogs; n = 17 thrombocytopenic dogs (13 primary IMT, 4 secondary IMT) + 20 healthy dogs for storage evaluation	Experimental/ clinical study	Flow cytometry	Reticulated platelets (%), absolute RP, PSAIgG	No diagnostic cut-off reported	RP% increased in **12/16 (75%)** thrombocytopenic dogs; absolute RP remained within reference values. RP% was stable up to 24 h, whereas PSAIgG increased with prolonged storage	Indirect	Small clinical cohorts; storage-related pre-analytical effects; RP findings were not consistently concordant with PSAIgG
**Maruyama et al., 2009 [2]**	Dogs; n = 20	Method-comparison study	Flow cytometry; thiazole orange staining; whole blood vs platelet-rich plasma	Reticulated platelets (%)	No diagnostic threshold reported	RP measurements in whole blood and platelet-rich plasma showed good agreement, supporting methodological comparability	Indirect	Primarily methodological; does not directly evaluate marrow activity
**Silva et al., 2012 [1]**	Dogs; n = 43 total (29 controls, 14 thrombocytopenic)	Experimental/ reference-standard study	Bone-marrow cytology + reticulated platelet measurement	RP%; bone-marrow megakaryocyte count	No diagnostic threshold reported	RP measurements were directly compared with bone-marrow megakaryocyte quantification; no significant correlation was demonstrated.	Direct	Direct marrow comparison was performed, but the study did not demonstrate a significant RP–megakaryocyte correlation.
**Oellers et al., 2016 [4]**	Dogs; n = 362 measurements overall; n = 193 healthy dogs for RI estimation (153 Beagles, 40 non-Beagles)	Reference-range/ analytical study	Sysmex XT-2000iV; manual gating	Reticulated platelets (r-PLT)	r-PLT RI: 0.0–1.2% (Pankraz-gate), 0.2–3.7% (Gelain-gate), and 0.2–3.9% (Oellers-gate)	The optimized Oellers-gate minimized interference from small erythrocytes/reticulocytes; agreement among gating strategies was high (rs = 0.88–1.00).	Indirect	Retrospective methodology; analyzer- and gating-specific measurement; interference from small erythrocytes/reticulocytes affected previously published gates; no direct bone-marrow reference standard.
**Smith & Thomas, 2002 [18]**	Dogs; sample size not reported.	Cross-sectional/ assay-development study	Flow cytometry; thiazole orange + anti-CD61	Reticulated platelets (%) and absolute RP	Healthy RI: **0–4.3%** and **0–12,095/µL**	No significant difference in RP% or absolute RP between healthy and clinically diseased nonthrombocytopenic dogs; established baseline for clinical use	Indirect	Primarily assay/reference-interval study; no direct marrow assessment
**Pankraz et al., 2009 [19]**	Dogs; n = 58 (40 healthy, 12 thrombocytopenic, 6 non-thrombocytopenic dogs with disorders associated with increased thrombopoiesis)	Method-comparison/ analytical validation study	FACScan flow cytometry with CD61-PE and thiazole orange; Sysmex XT2000iV PLT-O channel	r-PLT(F) and r-PLT(S)	r-PLT(F) RI: 0.78–3.68%; r-PLT(S) RI: 0.11–2.16%. Sysmex cutoff: ≥ 0.975%; sensitivity 94.74%, specificity 85.71%, LR + 6.63, LR − 0.06	r-PLT was significantly higher in the patient group than controls by both methods; flow cytometry and Sysmex showed fair correlation (r = 0.71). The automated method detected increased thrombopoiesis, but systematic bias between methods was observed.	Indirect	Method-comparison study using flow cytometry as reference method; analytical bias between platforms and sample-storage effects may affect results
**Kuhn et al., 2023 [5]**	Dogs; n = 41 for method validation (41 residual blood samples: 12 healthy, 29 diseased, including 22 thrombocytopenic); n = 120 healthy dogs for RI establishment	Analytical/methodological study	ADVIA 2120; moving-threshold gating	Reticulated platelets	Reference intervals were established for r-PLT measured using the ADVIA 2120 moving-threshold method; no clinical diagnostic cut-off was established.	The moving-threshold method showed strong agreement/correlation with flow cytometry but demonstrated a positive proportional bias; the method was evaluated for analytical performance and canine reference intervals rather than clinical diagnostic accuracy.	Indirect	Analyzer- and gating-specific methodological study; no direct marrow validation

^a^Abbreviations: IPF, immature platelet fraction; IPF#, absolute immature platelet fraction; PLT-F, platelet fluorescence channel; RP, reticulated platelets; r-PLT, reticulated platelets; RI, reference interval; CEN, central thrombocytopenia; PER, peripheral thrombocytopenia; PSAIgG, platelet surface-associated immunoglobulin G; BM, bone marrow.

^b^BM relationship: Direct indicates that the study included a direct bone-marrow reference standard; indirect indicates that the study provided evidence relevant to platelet production or thrombopoietic interpretation without direct bone-marrow examination.

Most studies used fluorescence-based methods, including Sysmex XN-1000V and XT-2000iV analyzers or thiazole-orange-based flow cytometry. One study evaluated reticulated platelets using the ADVIA 2120 analyzer. Only Silva et al. [1] incorporated a direct bone-marrow reference standard based on megakaryocyte cytology; importantly, this study did not demonstrate a significant correlation between reticulated platelet measurements and megakaryocyte counts. The remaining studies provided indirect evidence relevant to platelet production or thrombocytopenia through clinical classification, reference-interval establishment, analytical validation, or method comparison, without direct bone-marrow examination. The available evidence was therefore heterogeneous with respect to species, platelet parameter, analytical platform, study population, reference standard, and outcome measures. These differences were considered when interpreting the evidence and when assessing the feasibility of future quantitative evidence synthesis (Table 3).

### 3.4. Synthesis by species

#### 3.4.1. Dogs.

Reference intervals and/or analytical approaches for IPF or reticulated platelet parameters have been reported for the Sysmex XN-1000V [3, 15], Sysmex XT-2000iV [4, 19], and ADVIA 2120 [5], as well as for flow-cytometric methods [1, 2, 17, 18].Clinically, reticulated platelets have been analyzed in immune-mediated thrombocytopenia [16] and breed-related asymptomatic thrombocytopenia [17] – and One study directly compared reticulated platelet measurements with bone-marrow megakaryocyte counts; however, no significant correlation was demonstrated, indicating that direct biological validation of reticulated platelets as a surrogate marker of marrow megakaryocytic activity remains unresolved [1].

Taken together, the canine literature is broader and more methodologically diverse, encompassing reference-interval studies, analytical validation, method-comparison studies, clinical investigations, and one study incorporating a direct bone-marrow reference standard [1, 19].

#### 3.4.2. Cats.

Feline evidence is limited to a single study, in which IPF was measured on the Sysmex XN-1000V in 726 cats alongside 3281 dogs [15]. The feline reference interval (1–40.3%) was markedly wider than the canine interval (0.5–8%), and a cut-off below 13.6% was proposed to flag central thrombocytopenia after excluding platelet-clumping artifact. Receiver operating characteristic analysis suggested that IPF could discriminate central from peripheral thrombocytopenia in cats. However, the very wide reference interval implies substantially greater overlap between healthy and affected individuals than is seen in dogs, which may complicate single-value clinical interpretation.

### 3.5. Summary of the evidence gap

Of the 35 records identified, 27 unique records underwent title and abstract screening. Ten records were excluded at this stage, and 17 full-text reports were assessed for eligibility. Seven full-text reports were subsequently excluded, leaving 10 original studies for inclusion. Nine of the 10 included studies reported data exclusively in dogs, while one study included both dogs and cats; no feline-only study met the eligibility criteria. Only one included study directly evaluated a bone-marrow reference measure, consisting of megakaryocyte quantification, and this study did not demonstrate a significant correlation with reticulated platelet values. Importantly, the study using direct bone-marrow cytology did not demonstrate a significant correlation between reticulated platelet measurements and megakaryocyte counts. [1] Across the included studies, analyzer platforms differ considerably (Sysmex XN-1000V, Sysmex XT-2000iV, ADVIA 2120, flow cytometry), making direct comparison between studies difficult.

## 4. Discussion

### 4.1. Principal findings

The canine literature currently provides a broader and more methodologically diverse evidence base, including reference-interval studies, analytical validation and method-comparison studies, clinical investigations, and one study with a direct bone-marrow reference standard. This asymmetry may be due to several reasons, such as the fact that it may be harder to obtain adequate blood volumes from cats, the fact that platelets are known to clump in cats and may be more difficult to measure in an automated blood cell analyzer, and historically the higher clinical and research interest in canine immune-mediated thrombocytopenia.

### 4.2. Dogs versus cats: is one species better suited to IPF-based bone marrow assessment?

The evidence base for dogs is considerably larger than for cats, but current available data does not permit any conclusions to be drawn about superiority of one or both species regarding non-invasive assessment of bone marrow related platelet production with parameters measured by IPF or reticulated platelets as a biological marker. For canids many analytical techniques and differing clinical settings, and experimental designs have been applied to these markers, and in cats only one retrospective study has been carried out to assess the value of these markers [15]. There was a marked difference between reference intervals obtained in the study by Perez-Ecija et al. And the reference interval derived for cats [15]. There were specific criteria based on threshold levels proposed for central thrombocytopenia (based on species-specific values which differ from the respective reference intervals) although interpretation of the species specific reference interval differences should not be assumed to be purely a biological difference due to the ability of the canine to respond better to IPFs than the feline due to population characteristics, analytical equipment and design [1]. Significant directly biological validation is lacking in either species. There was only one study (in dogs) which compared reticulated platelet counts to quantitation of megakaryocytes from bone marrow, and found no relationship [12]. There is a direct bone marrow comparison for cats. Thus the only significant species difference at present is the number and scope of studies, not necessarily biological appropriateness between dogs and cats for IPF related bone marrow parameters to be determined as greater in one species than the other [20]. Studies using reproducible techniques across species are needed.

### 4.3. Implications for evidence synthesis: scoping review versus systematic review/meta-analysis

The evidence map produced by this scoping review will inform the approach to future evidence synthesis. For an effective systematic review with quantitative meta-analysis, studies must have had sufficient study development to meet the requirements for methodologic and clinical comparability, usually including standardization of index test definitions, reference standard definitions, diagnostic threshold definitions and outcome measures. We have established that the available evidence to date has failed this condition.

First, although 10 original studies were included, only 1 animal-specific evidence based on the animal was retrospective, making it difficult to achieve significant species-specific conclusions from the limited number of studies, and preventing any quantitative meta-analysis from being completed for the animal. Secondly there was considerable heterogeneity identified across analytical platforms, measuring techniques, populations and reference standards, all limiting the ability to perform a comparative analysis of diagnostic estimates. Third, the only studies including a direct bone-marrow reference standard where megakaryocyte cytology was examined was a single study; the remaining studies examined indirect or clinical classifications. This is a lack of consistent evidence even for a comparative study on canine diagnostic testing of IPF and related platelet parameters, thus preventing the ability to perform effective meta-analysis of pooled diagnostic estimates of IPF in canines.

The current evidence map is successful in addressing the need for a scoping review of the breadth of evidence, how the evidence can be characterized, the gap in existing evidence and the current methodologic conditions limiting quantitative synthesis. Consequently, the small number of studies and heterogeneity is one finding of the evidence map and is not the initial reason for selecting this type of review. A systematic review with diagnostic-accuracy meta-analysis will be premature at this point in time. However, it will become practical as a greater number of studies become available; ideally these would consist of a larger and more numerous prospective animal specific sample, with standardized measuring apparatus and analytical platforms with thresholds being used, measured against a bone-marrow reference standard. From the current evidence map we can prioritize further animal research to enable appropriate methodologic planning for a systematic review and meta-analysis.

### 4.4. Limitations

We searched four bibliographic databases; (PubMed/MEDLINE, Web of Science Core Collection, Scopus and CAB Abstracts). Searches may have missed evidence from non-indexed conference proceedings, grey literature and sources not in English.

All retrieved studies were searched full-text if possible. When individual components were not presented within the full paper, then a “not reported” status was recorded in lieu of estimation from the abstract. Since the purpose of the evidence-mapping approach for this scoping review was not to form a critical appraisal or a pooled diagnostic accuracy, risk-of-bias such as a QUADAS-2 assessment has not been utilized.

### 4.5. Future research directions

Priority should be given to prospective, multicenter feline studies that pair IPF/reticulated platelet measurement with a direct bone marrow reference standard (aspirate cytology or core biopsy), analogous to the canine study by Silva et al. Standardization of analyzer platform, gating strategy, and diagnostic cut-off definitions across studies – in both species – would substantially improve comparability and would be a prerequisite for any future diagnostic-accuracy meta-analysis.

## 5. Conclusion

While potentially there is valuable non-invasive information about thrombopoiesis and platelet generation in dogs and cats from IPF and Reticulated platelet parameters; as such these values cannot be put forth as established surrogate markers for bone-marrow-driven activity based on current evidence. This is especially true for cats, where it is limited to the one study included, but also for dogs due to the limited evidence base. Across included studies, there was significant heterogeneity between analytical platforms, platelet parameters measured, patient demographics and the standards used for diagnosis (i.e., diagnostic thresholds, reference methods). Critically, only one study compared reticulated platelet measurement with bone-marrow megakaryocytes directly and failed to find an association.

The evidence maps thus concluded that current literature lacked sufficient consistency and development for any meaningful quantitative synthesis or diagnostic accuracy meta-analysis. Future research needs to focus on prospective studies, especially on cats, and on measuring bone-marrow measurements as reference standards, using comparable analytical techniques and well-characterized populations to establish clinically significant cross-species or species-specific recommendations for IPF or reticulated platelet parameters.

## Supporting information

S1 File Excel file containing the search strategies for each database searched (PubMed/MEDLINE, WoS Core Collection, Scopus, and CAB Abstracts), the studies excluded at the full-text eligibility screening stage with the associated rationale, and the data extraction tables/forms including study characteristics, platelet parameters, diagnostic statistics, relevant observations, and study limitations. (XLSX)

S2 File PRISMA-ScR Checklist. Completed PRISMA Extension for Scoping Reviews (PRISMA-ScR) checklist for this scoping review.
(DOCX)
