# Supplementary material for: Immature platelet fraction and reticulated platelet parameters as potential indicators of bone-marrow-related platelet production in dogs and cats: A scoping review
Source: PLoS One. 2026 Sep 30;21(9):e0358333. doi: 10.1371/journal.pone.0358333 (PMC13626323; doi:10.1371/journal.pone.0358333)
Supplement: Supplementary file 1 [file pone.0358333.s002.docx]

PRISMA-ScR Checklist

*IPF/Reticulated Platelets in Dogs and Cats: A Scoping Review — completed against the submitted manuscript*

| **Reported on section/page** | **#** | **Checklist item** | **Reported on section/page** | **Status** |
| --- | --- | --- | --- | --- |
| **TITLE** |  |  |  |  |
| 1 | 1 | Identify the report as a scoping review. | *Title page — "...A Scoping Review"* | Reported |
| **ABSTRACT** |  |  |  |  |
| 2 | 2 | Provide a structured summary (background, objectives, eligibility criteria, sources of evidence, charting methods, results, conclusions). | *Abstract* | Reported |
| **INTRODUCTION** |  |  |  |  |
| 3 | 3 | Describe the rationale in context of what is already known; explain why a scoping review approach was chosen. | *Section 1, Introduction* | Reported |
| 3,4 | 4 | Provide an explicit statement of questions/objectives with key elements (population, concept, context). | *Section 1, end of Introduction* | Reported |
| **METHODS** |  |  |  |  |
| 4 | 5 | Indicate whether a review protocol exists; state where it can be accessed; provide registration information/number. | *Section 2.1 — Protocol registered at Zenodo (DOI: 10.5281/zenodo.21262147; retrospective registration disclosed).* | Reported (Zenodo DOI; retrospective registration disclosed) |
| 5 | 6 | Specify eligibility criteria (e.g., years, language, publication status) with rationale. | *Section 2.2* | Reported |
| 5 | 7 | Describe all information sources (databases, dates of coverage, contact with authors) and date of most recent search. | *Section 2.3 — literature search conducted through July 2026 (most recent/final search date: July 2026); database coverage dates as specified in search string* | Reported — final search date now specified (search updated through July 2026) |
| 5 | 8 | Present full electronic search strategy for at least one database, repeatable. | *Section 2.3 (search string)* | Reported |
| 6 | 9 | State the process for selecting sources of evidence (screening and eligibility). | *Section 2.4* | Reported |
| 6 | 10 | Describe methods of charting data (forms used; independent/duplicate charting). | *Section 2.5* | Partially reported — single-reviewer charting; not duplicated (noted as limitation) |
| 6 | 11 | List and define all variables for which data were sought. | *Section 2.5* | Reported |
| - | 12* | (Optional) Critical appraisal of individual sources of evidence — rationale and methods, if done. | *Not conducted (consistent with scoping review methodology)* | Not applicable (optional item) |
| 6 | 13 | Describe methods of handling and summarizing charted data. | *Section 2.5, last sentence* | Reported |
| **RESULTS** |  |  |  |  |
| 6,7 | 14 | Give numbers of sources screened, assessed, and included, with reasons for exclusion at each stage, ideally with a flow diagram. | *Section 3.1 + Figure 1 (PRISMA flow diagram)* | Reported |
| 8,9 | 15 | For each source, present charted characteristics and citations. | *Table 3 (charting table) + reference list* | Reported |
| - | 16* | (Optional) Present critical appraisal data, if done. | *Not conducted (optional item)* | Not applicable (optional item) |
| 9,10 | 17 | For each included source, present relevant charted data related to review questions. | *Table 3 + Section 3.4 narrative* | Reported |
| 10,11 | 18 | Summarize/present charting results as they relate to review questions and objectives. | *Section 3.4 (synthesis by species) + 3.5 (summary)* | Reported |
| **DISCUSSION** |  |  |  |  |
| 11,12 | 19 | Summarize main results (overview of concepts/themes/evidence types); link to objectives; consider relevance to key groups. | *Section 4.1-4.3* | Reported |
| 12,13 | 20 | Discuss limitations of the scoping review process. | *Section 4.4* | Reported |
| 13 | 21 | Provide general interpretation of results relative to objectives; implications/next steps. | *Section 5, Conclusion + 4.5 (future directions)* | Reported |
| **FUNDING** |  |  |  |  |
| 13 | 22 | Describe sources of funding for included sources of evidence and for the review itself; role of funders. | *Funding, Acknowledgments, and Conflicts of Interest sections (present, end of manuscript): "Funding: The present review was not externally funded. No funding was obtained for any of the included evidence sources." "Acknowledgments: The authors conducted this scoping review without external funding from inception until July 2026." "Conflicts of Interest: The authors declare no competing interests."* | Reported — no external funding; no competing interests declared |

**Legend:**

Green = fully reported	|	Yellow = partially reported (recommend strengthening) |	Red = missing (must add before submission) |	*Item 12 and 16 are the 2 optional items in PRISMA-ScR (critical appraisal), not required for scoping reviews.

*Source: Tricco AC, Lillie E, Zarin W, et al. PRISMA Extension for Scoping Reviews (PRISMA-ScR): Checklist and Explanation. Ann Intern Med. 2018;169(7):467-473. doi:10.7326/M18-0850.*
